# Creating ‘Partnership in iSupport program’ to optimise family carers’ impact on dementia care: a randomised controlled trial protocol

**DOI:** 10.1186/s12913-022-08148-2

**Published:** 2022-06-10

**Authors:** Lily Xiao, Ying Yu, Julie Ratcliffe, Rachel Milte, Claudia Meyer, Michael Chapman, Langduo Chen, Shahid Ullah, Alison Kitson, Andre Queiroz De Andrade, Elizabeth Beattie, Henry Brodaty, Sue McKechnie, Lee-Fay Low, Tuan Anh Nguyen, Craig Whitehead, Bianca Brijnath, Ronald Sinclair, Diana Voss

**Affiliations:** 1 College of Nursing and Health Sciences, Flinders University, Bedford Park, South Australia 5042 Australia; 2 Bolton Clarke Research Institute, Melbourne, VIC Australia; 3 Adjunct Research Fellow; Rehabilitation, Ageing and Independent Living Research Centre, Monash University, Melbourne, Australia; 4 Honorary Associate, Centre for Health Communication and Participation, La Trobe University, Melbourne, Australia; 5 Canberra Health Services, ACT, Canberra, Australia; 6 Southern Adelaide Local Health Network, Adelaide, South Australia Australia; 7 College of Medicine and Public Health, Flinders University, Bedford Park, South Australia Australia; 8 Quality Use of Medicines and Pharmacy Research Centre, Clinical and Medical Sciences, University of South Australia, Adelaide, Australia; 9 Queensland Dementia Training Study Centre, School of Nursing, Queensland University of Technology, Brisbane, QLD Australia; 10 Centre for Healthy Brain Ageing (CHeBA), School of Psychiatry, University of New South Wales, Sydney, NSW Australia; 11 Community Services, Resthaven Incorporated, Wayville, South Australia Australia; 12 Faculty of Health Sciences, University of Sydney, Sydney, Australia; 13 Social Gerontology Division, National Ageing Research Institute, Melbourne, Australia; 14 School of Allied Health, Curtin University, Bentley, West Australia Australia; 15 Faculty of Sciences, University of Adelaide, Adelaide, Australia

**Keywords:** Carers, Community aged care, Cost-effectiveness, Dementia, Health services research, Knowledge translation, Online education, Quality of life, Randomised controlled trial, Virtual social support

## Abstract

**Background:**

The majority of people with dementia are cared for by their family members. However, family carers are often unprepared for their caring roles, receiving less education and support compared with professional carers. The consequences are their reduced mental and physical health and wellbeing, and that of care recipients. This study protocol introduces the ‘Partnership in iSupport program’ that includes five interventional components: managing transitions, managing dementia progression, psychoeducation, carer support group and feedback on services. This health services research is built on family carer and dementia care service provider partnerships. The aims of the study are to evaluate the effectiveness, cost-effectiveness and family carers’ experiences in the program.

**Methods:**

A multicentre randomised controlled trial will be conducted with family carers of people living with dementia from two tertiary hospitals and two community aged care providers across three Australian states. The estimated sample size is 185 family carers. They will be randomly assigned to either the intervention group or the usual care group. Outcomes are measurable improvements in quality of life for carers and people with dementia, caregiving self-efficacy, social support, dementia related symptoms, and health service use for carers and their care recipients. Data will be collected at three time points: baseline, 6 months and 12 months post-initiation of the intervention.

**Discussion:**

This is the first large randomised controlled trial of a complex intervention on health and social care services with carers of people living with dementia in real-world practice across hospital and community aged care settings in three Australian states to ascertain the effectiveness, cost-effectiveness and carers’ experiences of the innovative program. We expect that this study will address gaps in supporting dementia carers in health and social care systems while generating new knowledge of the mechanisms of change in the systems. Findings will strengthen proactive health management for both people living with dementia and their carers by embedding, scaling up and sustaining the ‘Partnership in iSupport program’ in the health and social care systems.

**Trial registration:**

The Australian New Zealand Clinical Trials Registry (ANZCTR). ACTRN12622000199718. Registered February 4^th^, 2022.

**Supplementary Information:**

The online version contains supplementary material available at 10.1186/s12913-022-08148-2.

## Background

There were 55.2 million people living with dementia (PLWD) worldwide in 2019 and this number will reach 139 million in 2050 [1]. Up to 80% of PLWD also live with two or more additional chronic diseases [2]. People with dementia have more hospital admissions (1.4—4 times), more emergency care uses [2] and longer hospital stays [3] compared to those without dementia. They also frequently experience transitional care across care settings and care service providers due to complex care needs and are particularly vulnerable to gaps in health care and social care services (i.e. the lack of coordination between care settings and care providers) due to their high dependency on others to provide care [4]. Inadequate care and management of health conditions for this vulnerable population pose a great burden and high costs to health care and social care systems [5].

Family carers are the cornerstone of helping PLWD remain healthy, reducing unplanned hospital admissions and enabling them to stay at home for as long as they wish [1]. However, family carers receive less dementia education compared with professional carers to manage dementia and other complex health issues for PLWD [6] and receive limited ongoing support. Health services research that incorporates evidence-based practice to enhance support for carers in both hospital and community care settings is scarce. This study protocol reports an innovative and complex intervention to address gaps in supporting dementia carers through health and social care systems.

Similar to other developed countries, Australia has an increasing ageing population many of whom are living with dementia. Currently, there are 487,500 people estimated to be living with dementia in Australia and over 1.6 million people involved in their care [7]. Care services for PLWD after diagnosis are highly fragmented and difficult for carers to navigate and utilise [8]. Many carers feel socially isolated due to time spent on care, stigma and a lack of quality social networks [9]. Carer stress and distress are widely reported and contribute to poor health and reduced quality of life (QoL) of carers and PLWD [10], often resulting in premature permanent admission to nursing home care for PLWD [11]. A recent study in the USA, using the National Inpatient Sample from 2012 to 2016, revealed that 40% of hospitalisations of PLWD were due to potentially preventable conditions, for example falls, injuries, dehydration, poisoning or uncontrolled chronic diseases [12], strongly suggesting that educating and supporting carers to manage dementia and disease progression can avoid emergency department use and hospital admissions.

We propose a ‘Partnership in iSupport program’ that enables hospitals and community aged care organisations to implement evidence-based dementia care services to strengthen support for family carers of PLWD while tackling current problems in dementia care. There are three key pillars comprising the program: (1) a psychoeducation program for carers to improve their knowledge and skills in managing dementia using the online Australian iSupport for Dementia program; (2) iSupport program facilitators appointed by participating organisations acting as link workers to assist carers to navigate, access and utilise care services to meet the holistic care needs of PLWD; and (3) virtual carer support groups to enhance socialisation and reduce social isolation for carers. A program based on these pillars is viewed as a complex intervention due to its flexibility in delivering new services in real-world practice and needs to consider the mechanisms of change, facilitating factors and recipients of the program in a given health and social care context [13, 14].

**Fig. 1:**
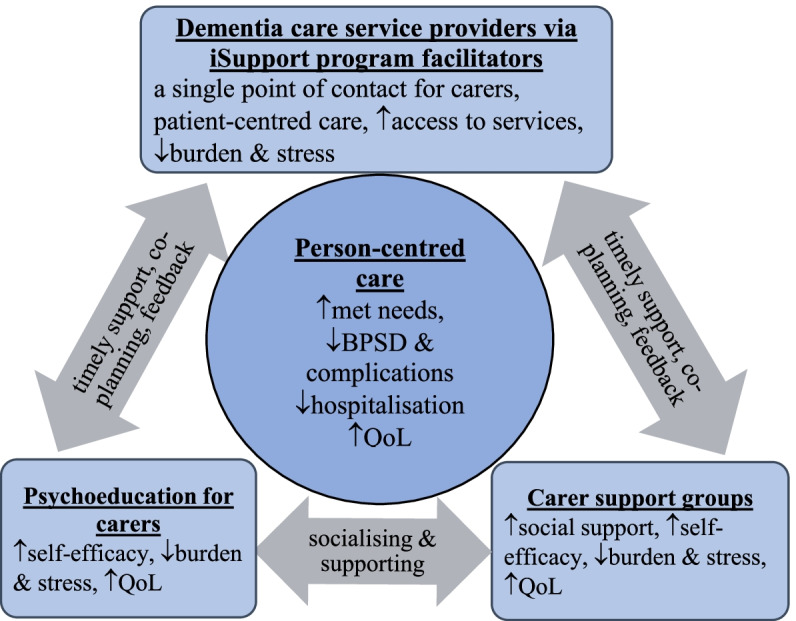
Partnership in iSupport program conceptual framework

Having a link worker who acts as a single point of contact for carers for a minimum of one year post-diagnosis support is a *standard* care service in Scotland [15]. Yet, this kind of support is viewed as an innovative dementia care model in a research paper submitted to the 2018–2020 Royal Commission into Aged Care Quality and Safety in Australia [16]. As noted in a recent Australian study, combining the three iSupport components in a single program reflects stakeholders’ expectations for providing a one-stop-shop for PLWD after diagnosis [8]. The program will use digital technology to improve reach to carers. The ‘Partnership in iSupport program’ conceptual framework is shown in Fig. 1 and discussed in the following sections.

### Psychoeducation and potential impact

Systematic reviews have determined that psychoeducation programs can significantly improve carers’ QoL and self-efficacy and reduce stress [17, 18]. The Australian iSupport program is a well-designed psychoeducation program for informal carers and is adapted from a generic version of the World Health Organization (WHO) iSupport for Dementia [19]. iSupport embeds holistic care, for example, providing opportunities for PLWD to choose and control activities which are meaningful, enjoyable and relevant to their life experiences. iSupport uses techniques, such as cognitive behavioural therapy, to help carers develop positive thoughts, problem-solving and coping skills, self-care and assertiveness when seeking help from relatives, friends and formal care service providers. The program covers dementia care from an early stage to the end of life. Therefore, it helps carers learn about changes as dementia progresses and take proactive action to gain timely treatment and care. When appropriate, the program introduces dementia care resources and multidisciplinary care services with weblinks for carers to access. The Australian iSupport program currently contains 6 modules and 30 units: introduction to dementia; being a carer; caring for me; providing everyday care; a person-centred care approach to changed behaviour; and my engagement in consumer directed care.

### iSupport program facilitators acting as link workers and potential impact

Two recent systematic reviews [17, 20] have confirmed that having a link worker to assist carers to navigate, access and utilise dementia care resources and multidisciplinary care services can significantly improve carers’ QoL and reduce their stress. A link worker is essential to bridge care gaps and prevent care crises during transitions between care settings and types [15]. In this study, iSupport program facilitators act as link workers.

### Virtual carer support groups and potential impact

Carer support groups can significantly reduce stress and improve self-efficacy and QoL for carers [1]. Video-streaming meetings facilitated by trained health professionals demonstrates better health outcomes for carers [21]. Virtual support groups enable carers to share their knowledge and experiences in dementia care, to learn from role models and to help each other [21]. Working carers particularly welcome virtual carer support as they can choose a convenient time to participate.

### Creating reciprocal partnerships to support person-centred care approach

iSupport program facilitators appointed by care service providers will assist carers to identify the individualised care needs of PLWD in a timely manner, especially when the PLWD’s health condition is deteriorating or during transitions between care settings (i.e. hospital-to-home) and care types (i.e. receiving palliative care at home). The facilitator will also assist carers to access and utilise relevant care services and co-plan care activities with carers and PLWD. It is anticipated that these supports will meet the care needs and improve QoL of PLWD, prevent and manage behavioural and psychological symptoms of dementia (BPSD) and complications; thus, reducing preventable conditions that contribute to admissions to emergency departments and hospitals for PLWD. Further, the carer support group activities enabled by facilitators in the study will create opportunities and empower carers to provide constructive feedback to care service providers regarding the strengths and weaknesses of services. Their feedback will inform service providers’ continuous quality improvement, organisational development and staff development in caring for people with dementia. Poor quality of care was identified by the recent Royal Commission into Aged Care Quality and Safety as a significant issue in Australia [22]. Only 20% of people receiving home care services felt their care needs were always met across all quality of care attributes [23]. The reciprocal partnerships built through the program will have high potential to mitigate these concerns and improve the quality of care.

### Study aims

This study is the second phase of a large project with three interrelated phases to emphasise a holistic approach to identify interventions with stakeholders, test evidence-based care services and translate knowledge into practice in a real-world setting. In phase 1, we worked with carers and care service providers to co-design the intervention to be delivered by iSupport program facilitators [24]. Phase 2 is this RCT. On the completion of the phase 2 study, we will translate the ‘Partnership in iSupport program’ into practice in participating organisations and disseminate the program nationwide (phase 3).

The aims of the present study are to (1) determine the intervention effectiveness; (2) establish the intervention cost-effectiveness; and (3) understand carers’ experiences in the program.

#### Primary hypotheses

Compared to those allocated to the usual care group, (1) carers receiving the intervention will report (a) at least a 5-point higher mean score on the mental component of the 12-Item Short-Form Health Survey (SF-12) and (b) an improved mean score on the physical component of SF-12; (2) their care recipients will report an improved mean score on the QoL in Alzheimer’s Disease (QOL-AD)-Proxy at 12-months follow-up.

#### Secondary hypotheses

Compared to those allocated to the usual care group, (1) carers in the intervention group will report (a) an improved mean score on the Caregiving Self-Efficacy Scale and (b) an improved mean score on the Quality of Social Support Scale; (2) their care recipients will report (carers acting as proxy respondents) (a) a reduced mean score on the Revised Memory and Behaviour Problem Checklist and (b) fewer unplanned hospital admissions, less preventable emergency department uses and less use of permanent residential aged care at 12 months; and (3) provision of the intervention will be more cost-effective than existing care.

## Methods/design

### Study design

A multicentre randomised controlled trial (RCT) with carers of PLWD will be conducted to test the primary and secondary hypotheses. The study protocol complies with the SPIRIT checklist [25], the CONSORT 2010 Checklist [26] and the TIDieR checklist [27] (see Supplementary File 1). The trial is registered on the Australian New Zealand Clinical Trials Registry (ANZCTR) website (see Supplementary File 2). The intervention will last 12 months. A cost effectiveness analysis will be undertaken alongside the RCT. A qualitative descriptive design will be applied to explore carers’ experiences in the program using data from virtual carer support group meetings. The across-method triangulation used in the study, via a combination of an RCT and a qualitative strand, will facilitate a more holistic understanding of iSupport program implementation [28].

### Sample size

The sample size calculation is based on the primary outcome of the SF-12 mental component for carers and is estimated on the basis of an earlier RCT with similar intervention components for carers [29]. In that study, the SF-12 mental component for carers increased significantly by an effect size (Cohen’s d) of 0.57 and standard deviation of 8.63. We assume that the same effect size would be observed in our trial. The estimated sample size would be 66 carers per arm using a 2-sample comparison of means for alpha = 0.05 at 90% of power. The Welch-Satterthwaite t-test is used to calculate the sample size for unequal variances. Assuming an attrition rate of 40%, the estimated sample size would be 185 carers in total. A Stata code power was used to calculate the sample size.

### Settings and participants

Participants will be family carers of PLWD who live at home. They will be recruited from four participating organisations (four study sites) as described in the following. We plan to recruit 46 carers (23 in each arm) in each of the four study sites. The organisations’ names are blinded to maintain confidentiality of information.

#### Hospital 1 and hospital 2

The study will be conducted in various care settings managed by two tertiary hospitals across two Australian states, for example in memory clinics, falls clinics for community dwelling PLWD, ambulatory geriatric services and geriatric/dementia wards where PLWD are ready to be discharged home and are expected to live at home for at least 12 months.

#### Aged care 1 and aged care 2

The study will be conducted in aged care settings where PLWD receive government subsidised aged care services at home provided by two large community aged care providers across two Australian states. The PLWD will have family carers to support them at home.

#### Inclusion criteria for carers

We will include carers who are (1) primary family carers aged 18 years or over; (2) providing care support for a person living with dementia at least twice a week; (3) the person with dementia lives at home and has mild to moderate dementia consistent with a score between 10 and 24 using the Mini Mental State Examination [30], and (4) the carer has access to the internet via a computer, a laptop or an iPad.

#### Exclusion criteria

Carers will be excluded if they (1) have health conditions that may significantly impact their ability to participate in the study; (2) are involved in other studies, and (3) cannot read English without additional assistance.

### Recruitment

Based on the inclusion and exclusion criteria, staff in the study sites will identify potential carer participants from their databases. Invitations to participate in the study with detailed information about the study will be sent to potential carers who will be asked to send a response sheet, email, SMS or voice message to a research associate indicating their interest in the study. The site-specific research associate will receive responses from potential participants and will approach them by phone or email to confirm their intention to participate. Once confirmed, the research associate will meet both the carer and PLWD either face-to-face or online to discuss the study in depth, answer any questions and take informed consent from carers and PLWD to perform the cognitive assessment for the PLWD to screen the carers’ eligibility to participate in the study.

### Data collection

The site-specific research associate will take another informed consent to participate in the study from carers and PLWD (who are capable of consenting for themselves) who have met the inclusion criteria and collect demographic information through a structured face-to-face interview. Carers will undertake a self-administered online survey at three time points: baseline, 6 months and 12 months post-initiation of the intervention. These surveys will help the research team to measure intervention effectiveness and cost-effectiveness. Carers will be notified about each survey by email and an SMS message sent by the research associate. The monthly carer support group meetings will be audio-recorded as data for the qualitative data analysis to understand carers’ experiences in the intervention.

### Randomisation

After baseline data collection, carers will be randomly assigned to receive either iSupport or usual care. To ensure the two groups are of equivalent size and conditions, a block randomisation will be used to allocate carers to one of the two treatment groups for each recruitment site. Blocks are determined by spouse caregivers versus non-spouse caregivers and care recipients with mild versus moderate dementia to ensure equivalent distribution of caregivers in each treatment group. An independent clinical trial centre based at the University of Sydney (https://www.ctc.usyd.edu.au/our-work/specialist-areas/randomisation/) will provide randomisation services to the study.

### The intervention group

The planned interventions and timelines are outlined in Table 1. iSupport program facilitators are trained nurses or social care professionals appointed by participating organisations.

**Table 1:**
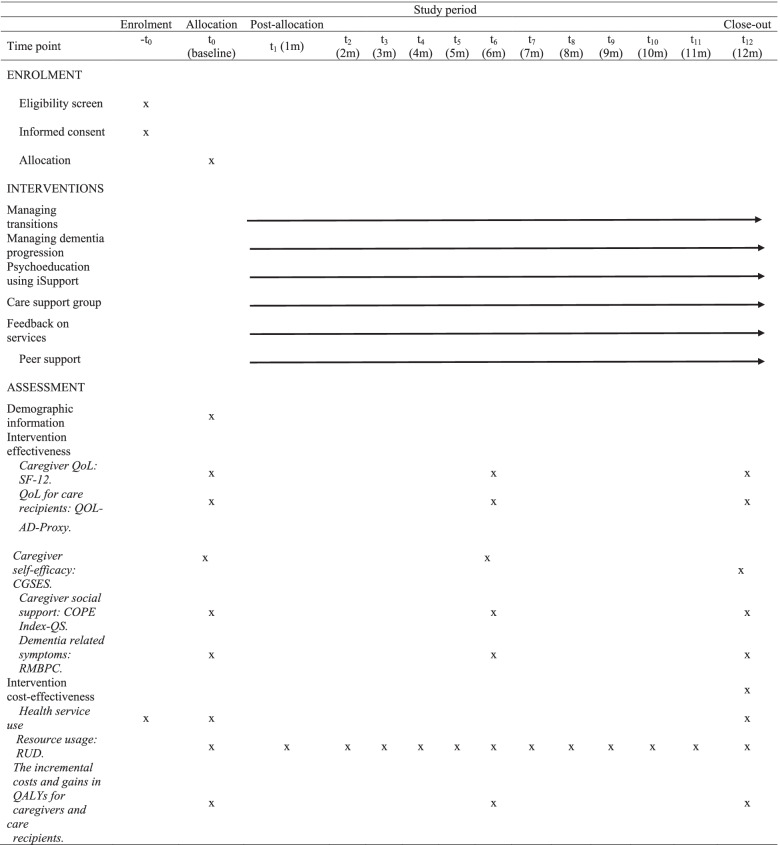
Time schedule of enrolment, interventions and assessment for participants

*Allocation after baseline data collection; QoL* Quality of Life, *SF-12* 12-Item Short-Form Health Survey, *QOL-AD-Proxy* Quality of Life in Alzheimer’s Disease-Proxy, *CSES* Caregiving Self-efficacy scale, *COPE Index-QS* Carers of Older People in Europe Index-Quality of Social Support, *RMBPC* Revised Memory and Behaviour Problem Checklist, Health service use: linked administrative health data from Data Linkage Services in Australia, *RUD* Resource Utilization in Dementia Lite Questionnaire which will measure unplanned hospital admissions, emergency department use and the use of permanent residential aged care for care recipients. *QALYs* Quality adjusted life years

#### Managing transitions

Carers will be assigned to an iSupport program facilitator and encouraged to request individualised support from the facilitator during transitions between care settings (i.e., from hospital to home transitional care) and types (i.e., receiving ambulatory geriatric services, or rehabilitation or palliative care at home in addition to usual home care services). Carers will make the request by phone or email. The individualised support for carers will be administered using phone or Zoom (an online meeting platform), which will be organised by the facilitator. The anticipated time spend on this type of support will be 1–2 h per week per carer or based on the carer’s needs and discretion. Provision of this type of support and the time the facilitator spends on supporting each individual carer will be recorded as part of data for analysis.

#### Managing dementia progression

Carers are also encouraged to contact the facilitator to discuss changes in the PLWD (i.e., signs of deterioration, changed behaviours and complications) and gain advice to access relevant multidisciplinary care services for timely treatment and care. The facilitator will follow the carers until their needs for support have been met. The anticipated time spent on this type of support will be 1–2 h per week per carer or based on the carer’s needs and discretion. The provision of this type of support and the time the facilitator spends on supporting each individual carer will be recorded as part of data for analysis.

#### Psychoeducation

Carers will select at least 20 out of 30 learning units that are relevant to them from the online iSupport program to complete over the first six months of the trial and revisit or learn new units when they feel it is needed in the second six months. The learning units include short readings, case studies and interactive activities using multiple choice questions and answers. The expected time spent on studying the online iSupport learning modules is no more than 12 h in total. The completion of each unit will be measured automatically through the program design and will be recorded as data for compliance with the intervention. Carers will receive a certificate for each unit they complete, a strategy to motivate them in the program.

#### Carer support group

In each study site, the facilitator will assign the 23 carers into one of two support groups. The facilitator will conduct monthly online carer support group meetings using the Zoom meeting platform and lasting no more than 30 min. Managing transitions between care settings and types and managing disease progression will be standard meeting agenda items so that carers can share their experiences and support each other in these dementia caregiving areas. The meetings will be audio recorded for carers in the same group to access. The facilitator will also create carer support groups in WhatsApp to encourage carers to talk or send text messages to their peers in the same group to strengthen social support. The facilitator will analyse group interactions, investigate carers’ needs for support and provide feedback to them. Attendance at monthly group meetings via Zoom, the messages shared by carers in chat groups and the amount of time each carer spends in a chat group via WhatsApp will be recorded as part of data for analysis.

#### Feedback on services

The facilitators will collect carers’ feedback via regular carer support meetings and discuss the feedback in their organisation’s usual quality improvement meetings.

### The usual care group

Carers in the usual care group will receive the usual carer support provided by Dementia Australia. They will receive a monthly reminder email that directs them to the Dementia Australia website where they can seek support if they so wish.

### Measures

The scheduled time points for assessing the outcomes are outlined in Table 1 and detailed in the following sections.

#### Social demographic information

We will collect socio-demographic information about carers and PLWD in the study at baseline only.

#### Intervention effectiveness

Data to measure intervention effectiveness will be collected at baseline, 6 months and 12 months post-initiation of the intervention.

*SF-12:* The SF-12 includes 12 items measuring two domains: mental health-related QoL and physical health-related QoL [31]. Construct validity is 0.92 for the mental component and 0.91 for the physical component. The test–retest reliability for the mental component is 0.76 and for the physical component is 0.89. Higher scores indicate better QoL.

*Care recipients’ Quality of Life in Alzheimer’s Disease (QOL-AD-Proxy):* This scale has 13 items. It has an internal consistency of 0.84–0.86 and test–retest reliability of 0.76–0.92 [32]. Higher scores indicate better QoL.

*Caregiving Self-Efficacy Scale:* This scale includes 3 subscales with 15 items: self-efficacy for obtaining respite, responding to atypical patient behaviours and controlling upsetting thoughts about caregiving [33]. It shows internal consistency of 0.8 and test–retest reliability of 0.70. Higher scores indicate better self-efficacy.

*Carer social support using* the *Carers of Older People in Europe Index-Quality of Social Support (COPE Index-QS):* This scale includes 5 items and shows an internal consistency of 0.76 and test–retest reliability of 0.80 [34]*.* Higher scores indicate better social support.

*Dementia related symptoms using the Revised Memory and Behaviour Problem Checklist (RMBPC)*: This scale includes 24 items measuring the frequency of memory and behaviour problems of PLWD and carers’ reaction to those problems in three subscales [35]: depression, disruption and memory-related problems. It has an internal consistency of 0.84 for frequency of behaviour and 0.90 for reaction to the behaviour and construct validity. Lower scores indicate less frequency of memory and behaviour problems and carers feeling less upset.

#### Intervention cost-effectiveness

*Health service use:* We will use linked administrative health data from Data Linkage Services in Australia, including Pharmaceutical Benefits Scheme (PBS) and Medicare Benefits Schedule (MBS) utilisation, and hospital and emergency department use data to measure health service use for carers and people with dementia. Data in these areas will be extracted 12 months prior and 12 months post-initiation of the intervention.

*Resource utilisation in dementia (RUD) questionnaire*: Health and social care visits of carers and people with dementia outside those provided by PBS, MBS and hospital data will be measured using the resource utilisation in dementia (RUD) questionnaire [36]. The data collection will be undertaken at baseline and then every month for 12 months to minimise recall bias during the 12-month trial using a self-administered online survey.

### Data analysis

#### Intervention effectiveness

A biostatistician who will be blinded to group assignments, will undertake data analysis. Data will be analysed on an intention-to-treat basis based on group assignments. Descriptive statistics will be applied to summarise data. Two-sample Student’s t test for baseline continuous variables with normal distribution, Wilcoxon rank-sum for non-normally distributed continuous variables, and Pearson’s χ^2^ test for categorical variables to explore any differences between the two groups will be conducted. A multivariate mixed effect linear regression model will be applied to fit linear mixed models to examine the primary and secondary outcomes between groups. As the outcome occurs for each individual with repeated time points, the mixed effect models will capture both fixed effects and random effects within the hierarchical structure of the data. The fixed effects, including group effect, time effect and group x time interaction, will be analogous to the regression coefficients. The random effects represent the estimated variability in the intercept to account for repeated measurements. Univariate models will be first used, then multivariate modelling will be undertaken by adding variables considered clinically important or statistically significant from the univariate model to adjust for confounding effects between variables. The model will also be adjusted by the baseline measure of outcome variable. The maximum likelihood estimate procedure will be used to compare significant differences in primary and secondary outcomes over time and between groups. A series of models will be undertaken by adding and subtracting variables, with changes in model fit assessed by log likelihood to choose the final multivariate model. The two-sided test will be performed for all analyses, 95% CI will be reported, and the level of significance will be set at *p* < 0.05. All analyses will be performed using Stata software version 16.1.

#### Intervention cost-effectiveness

The cost effectiveness analysis will be undertaken by a senior health economist. The primary measure of cost-effectiveness will be the incremental gains in quality adjusted life years (QALYs) from the perspective of carers and people with dementia compared to usual care. Resources associated with the development and implementation of the intervention will be documented and costed according to established best practice guidelines [37]. SF-12 responses between baseline and 12 months will be converted into health state utilities for the calculation of QALYs for carers using the SF-6D preference based scoring algorithm developed by Brazier et al [38]. QOL-AD-Proxy responses will be similarly converted using the preference based scoring algorithm developed by Comans et al [39]. Linked administrative health data from Data Linkage Services, including PBS and MBS utilisation, and hospital and emergency department uses, will be used to capture health service use for PLWD for 12 months prior and 12 months post recruitment with permission of the carers and their care recipients (when appropriate). Pre-baseline resource use data will provide additional baseline variables to control for potential confounding. The difference between pre- and post-baseline use of health services will be used to measure changes in PLWD’s levels of utilisation and cost of health services in both study arms. Data on health and social care visits outside those provided by MBS will be collected from participants using the Client Service Receipt Inventory [40]. Unit costs will be derived from hospital finance departments and Australian Refined Diagnosis Related Groups cost weights. Cost of nursing home care will be calculated using basic daily and accommodation fees apportioned according to length of stay. An assessment of the sensitivity of the results obtained to variation in measured resources, effectiveness and/or unit costs will be undertaken using appropriate one-way and multi-way sensitivity analysis [37, 38]. A budget impact analysis will also be conducted, extrapolating the within trial assessment of costs and outcomes to a population level [41].

#### Understanding carers’ experiences in the program

We will randomly select three audio-recorded carer support meetings per study site per 6 months to analyse carers’ experiences in the intervention. Audio data will be transcribed verbatim for analysis. Thematic analysis described by Nowell et al. will be applied [42]. An experienced qualitative researcher in the team will lead the data analysis.

### Implementation fidelity

A project implementation manual that details the aims, process, outcome measures, roles and responsibilities of personnel in the study will be developed to foster communication in the research team. A training program will be provided to facilitators and research assistants across the four study sites to standardise practice and compliance with the study protocol and ethics requirements. A written training manual will be developed to support the training program. The project manager will undertake monthly audits of the processes, compliances with planned intervention and carers’ satisfaction with the intervention. We will also monitor completion of online iSupport learning units by carers, consistency, satisfaction and quality of implementation across sites [43]. Each facilitator will document their key performances in the ‘Facilitators’ portfolio’ document and submit it monthly for audit. They will attend monthly meetings with the project team to discuss their activities.

### Data management and monitoring

We have gained ethics approval for planned data management and monitoring. The research team have relevant experiences in maintaining the integrity and security of the type of data we will collect for this study. The data will be verified and validated by two researchers to ensure the input is accurate. The research team will secure the data with limited system access. For example, during the data collection and data analysis period, raw data will be in electronic format, have unique codes for participants and be kept in password-protected electronic files. Only researchers in the project can access the data. De-identified online survey data in SPSS format and transcripts of the selected online carer support group meetings will be shared amongst the research team only. The hard copy data collected by each study site will be stored in a secure locked filing cabinet in a particular room in a particular building approved by the organisation of the study site. Only the principal investigator and the site-specific investigator can access the data to maintain proper governance. Data will be analysed collectively in the project team. Therefore, de-identified data stored on the secured cloud of Flinders University Research Drive, will be used to access and share the data between team members across multiple study sites during the study phase. To protect participants’ identity, all sensitive (i.e., audio-recordings) and demographic information will be stored on the Research Drive and no access will be given to the broader research team. Participants will be assured that pseudonyms will be used in reports and publications of the project.

We have established strategies and mechanisms approved by the relevant ethics committees to monitor and manage risks. We have established a Co-design Advisory Group which includes five carer and five professional representatives to review our regular reports on the study and advise on risk management or the modification of the study when needed. We will gain ethics approval for any modifications to the study and will inform the Trial Registry of the modifications. During the trial, the principal investigator will meet with program facilitators weekly to discuss any issues of concern they may have and address these concerns in a timely manner. Program facilitators will verbally remind carers in each carer group meeting to maintain confidentiality of information discussed in the group.

### Knowledge translation plan

The ‘Partnership in iSupport program’ that shows best effectiveness and cost-effectiveness in the study will be translated into routine services in new wards/units or new sites of hospitals and community aged care organisations participating in the study. We will disseminate the study nationally and invite hospitals and aged care organisations as new collaborators to roll out the ‘Partnership in iSupport program’. We will use research evidence to inform policy, resource and practice development to support the rollout of the program. Training programs for iSupport program facilitators and toolkits developed and tested in the study will enable the knowledge translation. Most importantly, the context of this study, organisational support for the program, and enablers and barriers identified from the study will be shared by organisations involved in the knowledge translation phase. We anticipate that this approach to knowledge translation will foster transparency, acceptance and effectiveness in health service interventions.

## Discussion

Ill mental health, such as depression and anxiety, and poor emotional wellbeing, described as stress, distress and emotional reactions to care, in carers of PLWD are widely reported [44, 45] and showed a detrimental impact on their quality of life [46, 47]. Moreover, these health conditions were associated with care recipients’ admissions to hospitals and emergency departments and permanent premature admissions to nursing home care [10, 48]. Preparing carers with capabilities to manage dementia and other chronic diseases for PLWD and supporting them to care for themselves are well-recognised strategies to maintain health, wellbeing and quality of life for both carers and PLWD, relieve burden on health and social care systems and reduce health costs [2, 5, 49]. Ironically, currently in health care and social care systems carers are not recognised as clients, nor receive support in managing dementia at a time they need while maintaining their own health [1]. We expect that this study will strengthen proactive health management for both the PLWD and their carers by providing carers with psychoeducation and individualised support at the time they need. Our program is in line with the call for interventions that target problems identified by stakeholders [14, 50, 51]. We anticipate that the ‘Partnership in iSupport program’ trialled in real-world practice along with carers’ experiences in the program will improve acceptability, adaptability and scalability in the knowledge translation phase of the project.

### The innovative aspects

It is well-recognised in implementation science that successful implementation of an evidence-based intervention into health care services depends on stakeholders’ views of the innovative aspects of the intervention [13, 52]. We have strong evidence through systematic reviews [2, 5, 17, 53] that the five intervention components described in our program are effective as they can significantly improve positive thoughts, subjective wellbeing, ability to manage dementia and social support. They also can reduce mental health problems and stress for carers and improve cost-effectiveness in dementia care services. A meta-analysis has also confirmed that a multicomponent intervention has the largest effects, compared to an intervention with a single component [17]. Therefore, the characteristics of our program comprise: (1) building the five intervention components into routine practice to address current problems; (2) in the early stage, or phase 1, of our project prior to the trial selecting existing evidence to use in the intervention with stakeholders to enhance users’ acceptability of the program [24] (3) offering new care services to address current problems; (4) aiming to measure the effectiveness, cost-effectiveness and carers’ experiences in the program; and (5) emphasising reciprocal-based partnerships between service providers and users through feedback on the quality of care [5, 15, 17]. Partnerships with clients in health and social care services are part of accreditation standards for Australian hospitals and aged care services [54, 55]. The innovative nature of the program is a significant attraction for industry partners and carers to be part of the study.

### Strong support from industry partners

Implementing a complex intervention in real-world practice requires strong support from industry partners in order to identify the mechanisms of change and the contextual factors affecting the change [13, 14]. In the ‘Partnership in iSupport program’, we have formed a team with chief investigators from each participating organisation as site-specific project leaders who have proven leadership on facilitating changes in health and social care services. Leadership is a core attributor to enable the implementation of evidence-based practice in an organisational context [56]. This strategically formed team not only ensures the organisations’ support for the program, but also creates an enabling environment in the participating organisations for program facilitators and carers to achieve planned intervention goals. Moreover, site-specific project leaders bring their expert knowledge about the organisational context (i.e., structures, policies, procedures, resources, staff and clients) that is necessary for the team to overcome operational issues, improve training and support for program facilitators and recruit carers in the program.

### Program facilitation

It is well argued in the Integrated Promoting Action on Research Implementation in Health Services framework (or i-PARIHS framework) that facilitators are interventionists who need to assess the contextual factors and develop strategies to enable participants to achieve intervention goals [13, 57]. It is also evident from a nationwide survey of UK dementia care networks that health professionals perceived lack of details and clarity when implementing the National Institute for Health and Care Excellence (NICE) Guidance in managing changed behaviours in PLWD [58]. To support program facilitators to implement the program, we explored detailed intervention activities that will be undertaken by facilitators and possible factors affecting their practice in phase 1 of the project [24]. We have also reviewed relevant literature on facilitation, education tools and other toolkits when implementing evidence-based practice [59, 60]. A tailored training program with tools and ongoing support for program facilitators has been planned to address issues of concern in program facilitation. In addition, we have nested a qualitative study to explore carers’ experiences in the program which will allow the project team to analyse contextual factors affecting program outcomes from carers’ perspectives. Such a research component is highly valued in a complex intervention to ascertain the mechanisms of change [14].

### Engagement with stakeholders

Successful health services research to implement evidence-based practice in a trial and beyond largely relies on ongoing engagement with stakeholders [14, 61]. We started to engage stakeholders at an early stage by inviting four carers and three staff representatives from the industry partners in the study to review a grant application for the study and by conducting phase 1 of the project to co-develop detailed intervention activities with stakeholders. Most importantly, we will embed the program into routine care services after the trial through the co-designed phase 3 of the project with stakeholders. In addition, the Co-design Advisory Group we formed in the project will continue to advise the project team about stakeholders’ perspectives of the program. These steps are key to ensure policy makers, dementia care service providers and carers accept the program and translate it into practice quickly.

## Supplementary Information

**Additional file 1.** CONSORT 2010 checklist of information to include when reporting a randomised trial*.

**Additional file 2:** **Supplementary File 2. **Information about trial registration data.

**Additional file 3.** Participant information sheet/consent form. 

## Data Availability

Not applicable.

## Abbreviations

PLWD: People living with dementia
RCT: Randomised controlled trial
