# Supplementary material for: Creating ‘Partnership in iSupport program’ to optimise family carers’ impact on dementia care: a randomised controlled trial protocol
Source: BMC Health Serv Res. 2022 Jun 10;22:762. doi: 10.1186/s12913-022-08148-2 (PMC9185883; doi:10.1186/s12913-022-08148-2)
Supplement: Supplementary file 2 — Additional file 2: Supplementary File 2. Information about trial registration data. [file 12913_2022_8148_MOESM2_ESM.docx]

**Supplementary File 2: Information about trial registration data**

| **Data category** | **Information** |
| --- | --- |
| Primary registry and trial identifying number | Australia New Zealand Clinical Trials Registry https://www.anzctr.org.au/Trial/Registration/TrialReview.aspx?id=380997&isClinicalTrial=False ; ACTRN12622000199718 |
| Date of registration in primary registry | 4 Feb, 2022 |
| Source of monetary or material support | National Health and Medical Research Council (Medical Research Future Fund [MRFF] 2020 Dementia Ageing and Aged Care grant and Dementia Collaborative Research Centre (2020 DCRC World Class Research Project Grants) |
| Primary sponsor | Australian Government |
| Contact for public queries | Professor Lily XIAO; Email: [lily.xiao@flinders.edu.au](mailto:lily.xiao@flinders.edu.au) |
| Contact for scientific queries | Lily XIAO, PhD, Professor, Flinders University, Australia |
| Public title | Creating ‘Partnership in iSupport program’ to optimise carers’ impact on dementia care |
| Scientific title | ‘Partnerships in iSupport program’ to optimise carers’ impact on dementia care: A randomised controlled trial |
| Settings of recruitment | Southern Adelaide Local Health Network, South Australia; Resthaven Inc. South Australia; Canberra Health Services, Australia; Bolton Clarke Victoria, Australia |
| Health conditions or problems studied | Dementia care |
| Interventions | Interventions: 1) managing transition, 2) managing dementia progression, 3) psychoeducation, 4) carer support group and 5) feedback to service |
|  | Control: the usual carer support provided by Dementia Australia or other publicly funded carer support. They will receive a monthly reminder email that directs them to the Dementia Australia website where they can seek support if they wish. |
| Key inclusion and exclusion criteria | Inclusion criteria: 1) The carer is aged 18 years or over; 2) The carer provides care support for a person living with dementia at least twice a week; 3) the person with dementia lives at home and has mild to moderate dementia consistent with a score between 10 and 24 using the Mini Mental State Examination, and 4) the carer has access to internet via a computer, or a laptop or an iPad. |
|  | Exclusion criteria: 1) have health conditions that may significantly impact their ability to participate in the study; 2) involve in other studies, and 3) cannot read English without additional assistance. |
| Study type | Interventional, randomized controlled trial, parallel, multicenter, data analysts blinded |
| Date of first enrolment | Anticipated: 30^th^ May 2022 |
| Target sample size | 184 |
| Recruitment status | Not started |
| Primary outcomes | 1. Caregiver quality of life; 2) Care recipients’ Quality of Life |
| Secondary outcomes | 1. Caregiver self-efficacy 2. Quality of social support for caregivers 3. Dementia related symptoms for care recipients 4. The incremental gains in quality adjusted life years (QALYs) for carers 5. The incremental gains in quality adjusted life years (QALYs) for people with dementia. 6. Health service use for carers and people with dementia 7. Health and social care visits of carer and people with dementia outside those provided by MBS |
