# Supplementary material for: Creating ‘Partnership in iSupport program’ to optimise family carers’ impact on dementia care: a randomised controlled trial protocol
Source: BMC Health Serv Res. 2022 Jun 10;22:762. doi: 10.1186/s12913-022-08148-2 (PMC9185883; doi:10.1186/s12913-022-08148-2)
Supplement: Supplementary file 3 — Additional file 3. Participant information sheet/consent form. [file 12913_2022_8148_MOESM3_ESM.docx]

**Participant Information Sheet/Consent Form**

| **Title** | Creating ‘Partnership in iSupport program’ to optimise family carers’ impact on dementia care: A randomised controlled trial protocol |
| --- | --- |
| **Protocol Number** | 2021/HRE00288 |
| **Coordinating Principal Investigator/ Principal Investigator** | Professor Lily Xiao |
| **Investigator(s)** | Professor Julie Ratcliffe; Dr Claudia Meyer; Dr Michael Chapman; Ms. Langduo Chen; Professor Alison Kitson; Dr Shahid Ullah; Dr Craig Whitehead; Ms Sue McKechnie; MS Ying Yu; Dr Rachel Milte; Dr Andre Queiroz De Andrade. |
| **Location** | The memory and geriatrics clinics, aged care and dementia care wards managed by Unit manager for Rehabilitation Ward, Southern Adelaide Local Health Network (SALHN) and Canberra Health Services; community aged care settings managed by Resthaven SA and Bolton Clarke Victoria. |

**Part 1 What does my participation involve?**

1. **Iintroduction**

You are invited to take part in this study, which is called “Creating ‘partnerships in iSupport program’ to optimise carers’ impact on dementia care”*.* You are invited to study if you have met all conditions listed below:

- You are an informal carer aged 18 years old or over.
- You provide care support for a person with dementia at least twice a week.
- The person with dementia whom you care for has mild to moderate dementia.
- You have access to internet via a computer, or a laptop or an iPad.

Please read this information carefully. Ask questions about anything that you don’t understand or want to know more about. Before deciding whether or not to take part, you might want to talk about it with a relative, friend or local health worker.

**Your participation is voluntary** (note: This section uses Services Australia prescribed.)

Your participation in this study is completely voluntary and there will be no cost to you or the person living with dementia you support and have legal guardianship/power of attorney status for. If you do not wish to take part in this study you do not have to. You should feel under no obligation to participate in this study. Choosing not to take part in this study will not affect your or the person you support’s current and future medical care in any way. You can withdraw your consent to participate in this study at any time.

If you decide to take part in the study, you will be asked to sign the consent section. By signing it you are telling us that you:

- understand what you have read;
- consent to take part in the study;
- consent to be involved in the research described;
- consent to the use of your personal and health information as described.

You will be given a copy of this Participant Information and Consent Form to keep.

**Your withdrawal from the study** (note: This section uses Services Australia prescribed wording.)

You are under no obligation to continue with the research study. You may change your mind at any time about participating in the research. People withdraw from studies for various reasons and you do not need to provide a reason.

You can withdraw from the study at any time by completing and signing the ‘**Participant Withdrawal of Consent Form**’. This form is provided at the end of this document, and is to be completed by you and supplied to the research team if you choose to withdraw at a later date.

If you withdraw from the study, you will be able to choose whether the study will destroy or retain the information it has collected about you. You should only choose one of these options. Where both boxes are ticked in error or neither box is ticked, the study will destroy all information it has collected about you.

1. **What is the purpose of this research?**

The study you are invited to participate in, is the second phase of a larger health service research project: “Creating ‘Partnership in iSupport program’ to optimise carers’ impact on dementia care”.

iSupport for Dementia program is a trustworthy, comprehensive online dementia education program for informal carers. It includes 6 learning modules: (1) introduction to dementia (5 units); being a carer (4 units); caring for me (3 units); providing everyday care (5 units); and person-centred care approach to changed behaviour (7 units) and (6) my engagement in consumer directed care (6 units). Each module presents a topic and provides interactive exercises with instant feedback for carers.

The ‘Partnership in iSupport program’ we will test in this study comprises not only the online education modules, but also services that will be delivered by an iSupport program facilitator to assist carers to navigate, access and utilise dementia care resources and care services; and to participate in online carer support groups.

The purpose of this part of the study is to work with carers and dementia care service providers to evaluate the outcomes of the ‘Partnership in iSupport program’ and to know carers’ experience in the program.

1. **What does participation in this study involve? What do I have to do?**

You will be participating in a trial. Sometimes we do not know which support is best for your situation. To find out we need to compare different support and programs. We put people into groups and give each group a different supporting program. The results are compared to see if one is better. To try to make sure the groups are the same, each participant is put into a group by chance (random). You will be randomly assigned to receive either the iSupport program or the usual care.

If you decide to take part in the study, the person you care for will be assessed by a researcher. This will determine if you are eligible to take part in the trial. Completing the assessment will take approximately 20 minutes. If the screening questionnaire shows that you meet the requirements, then you will be able to start the study. If the screening questionnaire shows that you cannot be in the study, the researcher will discuss other options with you.

After baseline data collection, you will be randomly assigned to receive either the iSupport program or the usual care. If you are in a usual care group, you will receive the usual carer support provided by Dementia Australia or other publicly funded carer support. You will receive a monthly reminder email that directs you to the Dementia Australia website where you can seek support if you wish. You will undertake an online survey at three time points: baseline, 6 months and 12 months. Each survey will take no more than 30 minutes. In addition, a researcher will contact you monthly to complete a 10-minute short survey on the usage of health care services and resources.

If you are in an intervention group:

- You will select at least 20 learning units that are relevant to you from the online iSupport website site to learn over 6 months. It will take no more than 12 hours in total to complete these units.
- A program facilitator will assign you into support groups of 6 people.
- The program facilitator will conduct a monthly carer support group meeting lasting no more than 30 minutes using Zoom meeting or mobile WhatsApp so that you can share your learning experiences and support each other. The meetings will be recorded for data analyses purposes and for carers in the same group to access late if they wish to do so.
- The program facilitator will create carer support groups using mobile WhatsApp to encourage each carer to talk or send text messages to your peers in the same group weekly to strengthen social support.
- The program facilitator will analyse group interactions and send a weekly short summary message to enhance carers’ performance in dementia care. Carers will follow an agreed protocol to maintain confidentiality and privacy in group activities.
- You will undertake an online survey at three time points: baseline, 6 months and 12 months. Each survey will take no more than 30 minutes.
- You will be contacted by a researcher to undertake a 10-minute short survey to know the health care resource use that you and the person you care for have had in the past month.
- You will be supported if you experience any technical issue.

There are no costs associated with participating in this study. You will receive $50 per month for 12 months to appreciate your time in the study.

1. **Other relevant information about the study**

***4.1. Medicare Benefits Schedule (MBS) and Pharmaceutical Benefits Scheme (PBS) Consent Form*** (note: This section uses Services Australia prescribed wording as it is required.)

You will be asked to sign a consent form authorising the study to access your complete Medicare Benefits Schedule (MBS) and/or Pharmaceutical Benefits Scheme (PBS) data as outlined in the consent form. In addition to consenting for the release of your own information you may be asked to be the third-party to sign the consent form for the person you support to allow Services Australia to release MBS/PBS information. Medicare collects information on your and the person you support’s doctor visits and the associated costs, while the PBS collects information on the prescription medications you and the person you support have filled at pharmacies. The consent form is sent securely to the Department of Human Services who holds MBS, PBS data confidentially.

***4.2. The study locations***

The study locations are (1) memory and geriatric clinics and aged care and dementia care wards managed by SALHN and Canberra Health Services; and (2) community aged care settings managed by Resthaven SA and Bolton Clarke Victoria. The Flinders University employed researcher will undertake screening, recruitment and collect demographic information of you and your care recipients that is relevant to this study. Demographic information is also known as personal information such as age, gender, education levels, employment, income and marriage status. We plan to recruit 184 carers in total in the study.

1. **What does it mean to provide consent to using my health information?**

SA Health/Department of Health and Human Services Victoria/ACT Government Health use personal and health information extracted from health records to run the health system. The health information exists in a number of State and Commonwealth administrative datasets and are de-identified to ensure your personal privacy is protected.

By supporting this research study, you are agreeing to the use of your and your care recipient’s health information as held in the administrative databases that have come from your or their health records. On behalf of the research team, SA NT Datalink/Victorian Centre for Data Linkage/NSW Centre for Health Record Linkage will link your health information from the following sources:

- Public and private hospital admissions, emergency departments, held by the SA Health/Department of Health and Human Services Victoria/ACT Government Health.

This will also be combined by the researchers with MBS records (i.e., visits to health professionals) and PBS records (i.e., use of prescription medicines) and other information we collect in the study (for example responses to questionnaires ask you to fill out).

The linked health information provided to the research team will be in a de-identified form. It will contain personal identification numbers and health information but no names, dates of birth or home addresses. Any health information used from these data sources are managed completely confidentially and are used only for the purpose of the research as described for this study. With your agreement, your and your care recipient’s health information (as drawn from health records in the administrative datasets listed above) will be included in the linked health information.

1. **To participate in the study, do I have to consent to linking my health information?**

No. If you want **to opt-out** of the linking of your health information, there is an option to indicate this choice on the consent form by ticking the box for opt-out.

1. **How can I express my interest in the study?**

There are a few ways to express your interest.

- When approached for expression of intention, you can verbally inform the researcher your intention to participate. The researcher will make an appointment with you for further discussion.
- You can text message or ring the researcher (Ms Ying Yu, Mobile phone number: 0412904168) to express your interest in participation.
- You can email the researcher to express your interest in participation (Ms Ying Yu, email: [ying.yu@flinders.edu.au](mailto:ying.yu@flinders.edu.au))

On receipt of your response, the researcher will contact you to confirm your participation. More detailed information related to the research will be explained to you by the researcher if you would like to know.

1. **What are the possible benefits of taking part?**

We cannot guarantee or promise that you will receive any benefits from this research. However, participants in the intervention group will select learning units from the online iSupport program that are relevant to them to learn. Each module presents a topic and provides interactive exercises with instant feedback for carers. When appropriate, the program introduces dementia care resources and services with weblinks for carers to access. The iSupport program empowers carers to seek help and gain practical solutions from the service provider and peers in a timely manner.

1. **What are the possible risks and disadvantages of taking part?**

The researchers do not expect the questions to cause any harm or discomfort to you. You may feel that some of the questions we ask are stressful or upsetting. If you do not wish to answer a question, you may skip it and go to the next question, or you may stop immediately. If you experience feelings of distress as a result of participation in this study, please let the research team know immediately. The research team will be able to arrange for counselling or other appropriate support. Any counselling or support will be provided by qualified staff who are not members of the research team. This counselling will be provided free of charge. You can also contact the following services for support:

Dementia Australia – 1800 100 500

Lifeline – 13 11 14, www.lifeline.org.au

Beyond Blue – 1300 22 4636, www.beyondblue.org.au

There is a small risk to your and your care recipient’s health information privacy because personal information is used in obtaining MBS and PBS records. We will provide personal information to the linking agencies (for example name, date of birth, and home address), so they can identify you or the person you care for in their databases, and make sure they only provide us with information about people who have consented to the study. We minimise any risk by providing this information directly to the linking agency in an encrypted and password protected file. This risk is further minimised by separating the processes of record linkage and data analysis. The record linkage only uses personal information such as name, date of birth, and home address. After we have gained the linked information, we will replace the personal information with a unique personal identification number.

The linked health information provided to the researchers contains personal identification numbers and health information but no names, dates of birth or home addresses. All privacy measures have been put in place to ensure that the confidentiality of your and the person you care for are maintained, including removal of identifying information, the use of unique study numbers and adherence to strict guidelines regarding data transfer, storage and access.

You may feel the program is time consuming. All activity in the research is self-paced. You can choose the time and date that are suitable for you to engage in the online iSupport program. You also have the rights to choose the time that is convenient for you to participate in the online carer support group meetings. If you still feel the time commitment is too great to complete a given component of the study, you may withdraw from the study at any point. You will receive $50 gift voucher per month for 12 months as a token of appreciation for your time contributing to the study.

1. **What happens when the study ends?**

After the study completed, a summary of the study findings will be sent to you by email or posting to you with your permission*.*

**Part 2 How is the study being conducted?**

1. **What will happen to information about me?**

***11.1. Storage, retention and destruction of your or the person your support’s information***

By signing the consent form, you consent to the research team collecting and using personal information about you or the person you care for. Any information obtained in connection with this study that can identify you or the person you care for will remain confidential. Any information provided by you will be collected in a de-identifiable form. Therefore, the data will not be linked to the individual or organisation. While we will also have identifiable information about you and the person you care for (for example your name, address, and Medicare number) to allow us to undertake the research, this will be kept separately from any data stored in de-identifiable form to reduce the risk of re-identifying you or your care recipient at any stage of the study. Hardcopy data collected through this research will be stored in a locked cabinet in the researcher’s office and electronic data will be kept on the Flinders University Research Drive (physically located in Australia), which is password protected. All study-related data will only be accessible to the researchers.

All data will be retained for 15 years, and then it will be destroyed. MBS/PBS data will not be used in any future or unspecified research outside of the approved study. Your and your care recipient’s information will only be used for the purpose of this study and it will only be disclosed with your permission, except as required by law. The linked health information as provided by the SA NT Datalink, Department of Health and Human Services Victoria or ACT Government Health will not be shared beyond the research team.

It is anticipated that the results of this research will be published and/or presented in a variety of forums. In any publications and/or presentations, information will be provided in such a way that you cannot be identified, except with your permission.

1. **Complaints and compensation**

There is no foreseen adverse event and outcomes associated with your participation in this project. For matters relating to research at the facility where you are participating in the project, you should contact the local complaints contact person: Professor Lily Xiao (College of Nursing and Health Sciences, Flinders University, Telephone: +61 8 82013419) or complaints contact in Ethical commitment of South Australia.

1. **Who is organising and funding this study?**

This study is being conducted by a team of researcher led by Professor Lily Xiao. The project is funded by the Australia Government via the Medical Research Future Fund 2020 Dementia Ageing and Aged Care Mission ($1,406,658) and the Dementia Collaborative and Research Centre World Class Research Project Grants ($ 536,586).

1. **Who has reviewed this study?**

All research in Australia involving humans is reviewed by an independent group of people called the Human Research Ethics Committee (HREC). The ethical aspects of this research have been approved by the HREC of the The Southern Adelaide Clinical Human Research Ethics Committee (SAC HREC).

This research will be carried out according to the National Statement on Ethical Conduct in Human Research (2007; updated in 2018). This statement has been developed to protect the interests of people who agree to participate in human research studies.

1. **Further information and who to contact**

The person you may need to contact will depend on the nature of your query. If you want any further information concerning this project or if you have any problems which may be related to your involvement in the project, you can contact the researcher or any of the following people:

**Research contact person**

| Name | Professor Lily Xiao |
| --- | --- |
| Position | Principal investigator |
| Telephone | +61 8 82013419 |
| Email | Lily.xiao@flinders.edu.au |

For matters relating to research at the site at which you are participating, the details of the local site complaints person are:

**Complaints contact person**

| Name | Southern Adelaide Local Health Network |
| --- | --- |
| Position | Manager, Research Governance and Ethics |
| Telephone | (08)82046453 |
| Email | Health.SALHNofficeforResearch@sa.gov.au |

If you have any complaints about any aspect of the project, the way it is being conducted or any questions about being a research participant in general, then you may contact:

| Reviewing HREC name | Southern Adelaide Clinical Human Research Ethics Committee |
| --- | --- |
| Position | HREC Executive Officer |
| Telephone | (08) 8204 6453 |
| Email | Health.SALHNofficeforResearch@sa.gov.au |

**Reviewing HREC approving this research** **and HREC Executive Officer details**

**Local HREC Office contact**

| Name | Southern Adelaide Local Health Network |
| --- | --- |
| Position | Research Governance Officer |
| Telephone | 8204 6453 |
| Email | Health.SALHNofficeforResearch@sa.gov.au |

**Consent Form -** *Adult providing own consent*

| **Title** | Creating ‘partnerships in iSupport program’ to optimise carers’ impact on dementia care |
| --- | --- |
| **Short Title** | iSupport study |
| **Protocol Number** | 2021/HRE00288 |
| **Project Sponsor** | NHMRC/DCRC World Class Research Project Grants |
| **Coordinating Principal Investigator/**  **Principal Investigator** | Professor Lily Xiao |
| **Investigator(s)** | Professor Julie Ratcliffe; Dr Claudia Meyer; Dr Michael Chapman; Ms. Langduo Chen; Professor Alison Kitson; Dr Shahid Ullah; Dr Craig Whitehead; Ms Sue McKechnie; MS Ying Yu; Dr Rachel Milte; Dr Andre Queiroz De Andrade. |
| **Location** | Memory and geriatric clinics, aged care and dementia care wards managed by Sothern Adelaide Local Health Network (SALHN) and Canberra Health Services. The community aged care of Resthaven SA; Bolton Clarke Victoria. |

**Declaration by Participant**

I (being over the age of 18 years) have read the Participant Information Sheet or someone has read it to me in a language that I understand.

I understand the purposes, procedures and risks of the research described in the project.

I have had an opportunity to ask questions and I am satisfied with the answers I have received.

I freely agree to participate in this study as described and understand that I am free to withdraw at any time during the project without affecting my future care.

I freely agree to audio recording of my information and participation in the carer support group meetings (iSupport program group only).

I understand that I will be given a signed copy of this document to keep.

I am aware that I should retain a copy of the Information Sheet and Consent Form for future reference.

I have had an opportunity to ask questions and I am satisfied with the answers I have received.

I understand that:

- I may not directly benefit from taking part in this research.
- I am free to withdraw from the research at any time and am free to decline to answer particular questions.
- While the information gained in this study will be published as explained, I will not be identified, and individual information will remain confidential.
- Whether I participate or not, or withdraw after participating, it will have no effect on any treatment or service that is being provided to me.
- While no identifying information will be published, given the nature of group discussion my anonymity cannot be guaranteed (iSupport program group only).
- While I may withdraw at any time I may not ask for the audio recording to be stopped (iSupport program group only).
- I understand that researchers use my identifiable person information to access and link information about any public and private hospital admissions, or emergency department admissions for myself during the trial period held by
  - SANT Datalink (via the SA Public Hospital Separations and SA Public Hospital Emergency Department) OR
  - Department of Health and Human Services Victoria (via the Victorian Admitted Episodes Dataset, and the Victorian Emergency Minimum Dataset) OR
  - ACT Government Health (via the ACT Admitted Patient Care and the ACT Emergency Department Data Collection)

so that they can understand the costs related to the study treatments. I understand that only the research team will have access to this information, and it will be de-identified prior to analysis.

|  | | | | | | | |
| --- | --- | --- | --- | --- | --- | --- | --- |
|  | Name of Participant (please print) | |  | |  |  |  |
|  | | | | | | | |
|  | Signature |  | | Date | |  |  |
|  | | | | | | | |

**Declaration by Researcher^†^**

I have given a verbal explanation of the study, its procedures and risks and I believe that the participant has understood that explanation.

|  | | | | | | |
| --- | --- | --- | --- | --- | --- | --- |
|  | Name of Researcher^†^ (please print) | |  | | |  |
|  | | | | | |  |
|  | Signature |  | | Date |  |  |
|  | | | | | | |

^†^ An appropriately qualified member of the research team must provide the explanation of, and information concerning, the study.

Note: All parties signing the consent section must date their own signature
